# Cardiac outcomes of subjects on adjuvant trastuzumab emtansine vs paclitaxel in combination with trastuzumab for stage I HER2-positive breast cancer (ATEMPT) study (TBCRC033): a randomized controlled trial

**DOI:** 10.1038/s41523-022-00385-2

**Published:** 2022-02-16

**Authors:** Romualdo Barroso-Sousa, Paolo Tarantino, Nabihah Tayob, Chau Dang, Denise A. Yardley, Steven J. Isakoff, Vicente Valero, Meredith Faggen, Therese Mulvey, Ron Bose, Jiani Hu, Douglas Weckstein, Antonio C. Wolff, Katherine Reeder-Hayes, Hope S. Rugo, Bhuvaneswari Ramaswamy, Dan Zuckerman, Lowell Hart, Vijayakrishna K. Gadi, Michael Constantine, Kit Cheng, Frederick Briccetti, Bryan Schneider, Audrey Merrill Garrett, Kelly Marcom, Kathy Albain, Patricia DeFusco, Nadine Tung, Blair Ardman, Rita Nanda, Rachel C. Jankowitz, Mothaffar Rimawi, Vandana Abramson, Paula R. Pohlmann, Catherine Van Poznak, Andres Forero-Torres, Minetta Liu, Kathryn J. Ruddy, Yue Zheng, Shoshana M. Rosenberg, Richard D. Gelber, Lorenzo Trippa, William Barry, Michelle DeMeo, Harold Burstein, Ann Partridge, Eric P. Winer, Ian Krop, Sara M. Tolaney

**Affiliations:** 1 Oncology Center, Hospital Sírio-Libanês Brasília, Brasília, Brazil; 2 European Institute of Oncology IRCCS, Milan, Italy; 3 University of Milan, Milan, Italy; 4 Dana-Farber Cancer Institute, Boston, MA USA; 5 Harvard Medical School, Boston, MA USA; 6 Memorial Sloan Kettering Cancer Center, New York, NY USA; 7 Sarah Cannon Research Institute and Tennessee Oncology, Nashville, TN USA; 8 Massachusetts General Hospital, Boston, MA USA; 9 The University of Texas MD Anderson Cancer Center, Houston, TX USA; 10 Washington University, St Louis, MO USA; 11 Johns Hopkins Sidney Kimmel Cancer Center, Baltimore, MD USA; 12 UNC Chapel Hill, Chapel Hill, NC USA; 13 UCSF, San Francisco, CA USA; 14 OSU Comprehensive Cancer Center, Columbus, OH USA; 15 St Luke’s Mountain States Tumor Institute, Boise, ID USA; 16 Wake Forest Baptist Health, Winston-Salem, NC USA; 17 University of Washington, Seattle, WA USA; 18 Vijayakrishna K. Gadi’s current affiliation is University of Illinois at Chicago, Chicago, IL USA; 19 North Shore-LIJ Cancer Institute, Lake Success, NY USA; 20 IU School of Medicine, Indianapolis, IN USA; 21 Northern Light Cancer Care, Brewer, ME USA; 22 Duke University, Durham, NC USA; 23 Loyola University Medical Center, Maywood, IL USA; 24 Hartford Healthcare Cancer Institute, Hartford, CT USA; 25 Beth Israel Deaconess Medical Center, Boston, MA USA; 26 Lowell General Hospital, Lowell, MA USA; 27 The University of Chicago, Chicago, IL USA; 28 Abramsom Cancer Center, University of Pennsylvania, Philadelphia, PA USA; 29 Dan L. Duncan Comprehensive Cancer Center, Baylor College of Medicine, Houston, TX USA; 30 Vanderbilt-Ingram Cancer Center, Nashville, TN USA; 31 Lombardi Comprehensive Cancer Center, Georgetown University Medical Center, Washington, DC USA; 32 Rogel Cancer Center, University of Michigan, Ann Arbor, MI USA; 33 Kirklin UAB Hematology Oncology, Birmingham, AL USA; 34 Mayo Clinic, Rochester, MN USA; 35 Weill Cornell Medicine, New York, NY USA

**Keywords:** Breast cancer, Breast cancer

## Abstract

The excellent outcomes seen in patients treated with adjuvant trastuzumab emtansine (T-DM1) in the ATEMPT trial and the favorable toxicity profile associated with this agent make T-DM1 a potential therapeutic option for select patients with stage I HER2-positive breast cancer. Moreover, T-DM1 is an established adjuvant treatment for patients with HER2-positive breast cancer with the residual invasive disease after neoadjuvant therapy. Given that cardiotoxicity is the most significant adverse event of trastuzumab, which is a main molecular component of T-DM1, we conducted a sub-analysis of the ATEMPT trial to determine the cardiac safety of adjuvant T-DM1. In this analysis, the incidence of grade 3–4 left ventricular systolic dysfunction (LVSD) in T-DM1 or trastuzumab plus paclitaxel arms were respectively 0.8 and 1.8%. In addition, three (0.8%) patients in the T-DM1 arm and six (5.3%) patients in the adjuvant paclitaxel with trastuzumab (TH) arm experienced a significant asymptomatic left ventricular ejection fraction (LVEF) decline that per-protocol required holding T-DM1 or trastuzumab. All patients with available follow-up data experienced full resolution of cardiac symptoms and LVEF normalization. Furthermore, we performed an exploratory analysis to assess the relationship between age, baseline LVEF, and body mass index with cardiac outcomes. No significant association between these baseline characteristics and the incidence of significant asymptomatic LVEF decline or symptomatic LVSD was identified. The low incidence of significant cardiac adverse events in this population during therapy with adjuvant T-DM1 suggests that studies on the cost-effectiveness of cardiac monitoring during adjuvant therapy using anthracycline-free regimens are needed.

**Clinical Trial Registration:** ClinicalTrials.gov, NCT01853748

## Introduction

Amplification or overexpression of the human epidermal growth factor receptor 2 (HER2/neu) oncogene is present in ~15–20% of early-stage breast cancers^1,2^, identifying an aggressive disease subtype with a relatively high risk of recurrence in the absence of HER2/neu-directed systemic therapy^3^. However, the development of trastuzumab and a wide variety of additional biologic agents targeting HER2/neu in the last two decades have provided great clinical benefits to this subgroup of patients, significantly improving long-term outcomes^4^.

While patients with stage I HER2-positive breast cancer were either excluded from or underrepresented in pivotal trials that established the survival benefits of trastuzumab in combination with poly-chemotherapy, retrospective data of untreated patients showed that even these small HER2-positive breast cancers have recurrence rates between 10–30%, justifying the need of adjuvant treatment in this context^5–8^.

Efforts to evaluate less toxic adjuvant regimens for small HER2-positive breast cancer have been conducted^9^, and based on the excellent efficacy outcomes in the adjuvant paclitaxel and trastuzumab (APT) study^10,11^, adjuvant paclitaxel with trastuzumab (TH) is currently considered a standard option for patients with stage I HER2-positive breast cancer^12,13^. More recently, data from the adjuvant trastuzumab emtansine versus paclitaxel in combination with trastuzumab for stage I HER2-positive breast cancer (ATEMPT) study demonstrated that, among patients with stage I HER2-positive breast cancer, 1 year of adjuvant T-DM1 was associated with a 3-year invasive disease-free survival rate of 97.8% (95% confidence interval [CI]: 96.3–99.3%)^14^. Furthermore, patient-reported outcomes indicated that patients treated with T-DM1 had less neuropathy and alopecia, and better work productivity compared with patients on TH, suggesting T-DM1 as an alternative treatment option for small HER2-positive tumors. Adjuvant T-DM1 is also an established treatment for patients with HER2-positive breast cancer not achieving pathological complete response after neoadjuvant therapy^15^. There has consequently been substantially more use of T-DM1 as adjuvant treatment, and subsequently more risk of T-DM1-related adverse events.

Since cardiotoxicity is the most significant adverse event of trastuzumab, which is a main molecular component of T-DM1, the objective of the current analysis is to determine the cardiac safety of T-DM1 among patients on the ATEMPT study. As ATEMPT is the only study in which patients with the early-stage disease received 1 year of T-DM1 without any other systemic chemotherapy, this is an ideal opportunity to assess the cardiac safety of T-DM1 monotherapy.

## Results

### Patient characteristics

**Fig. 1:**
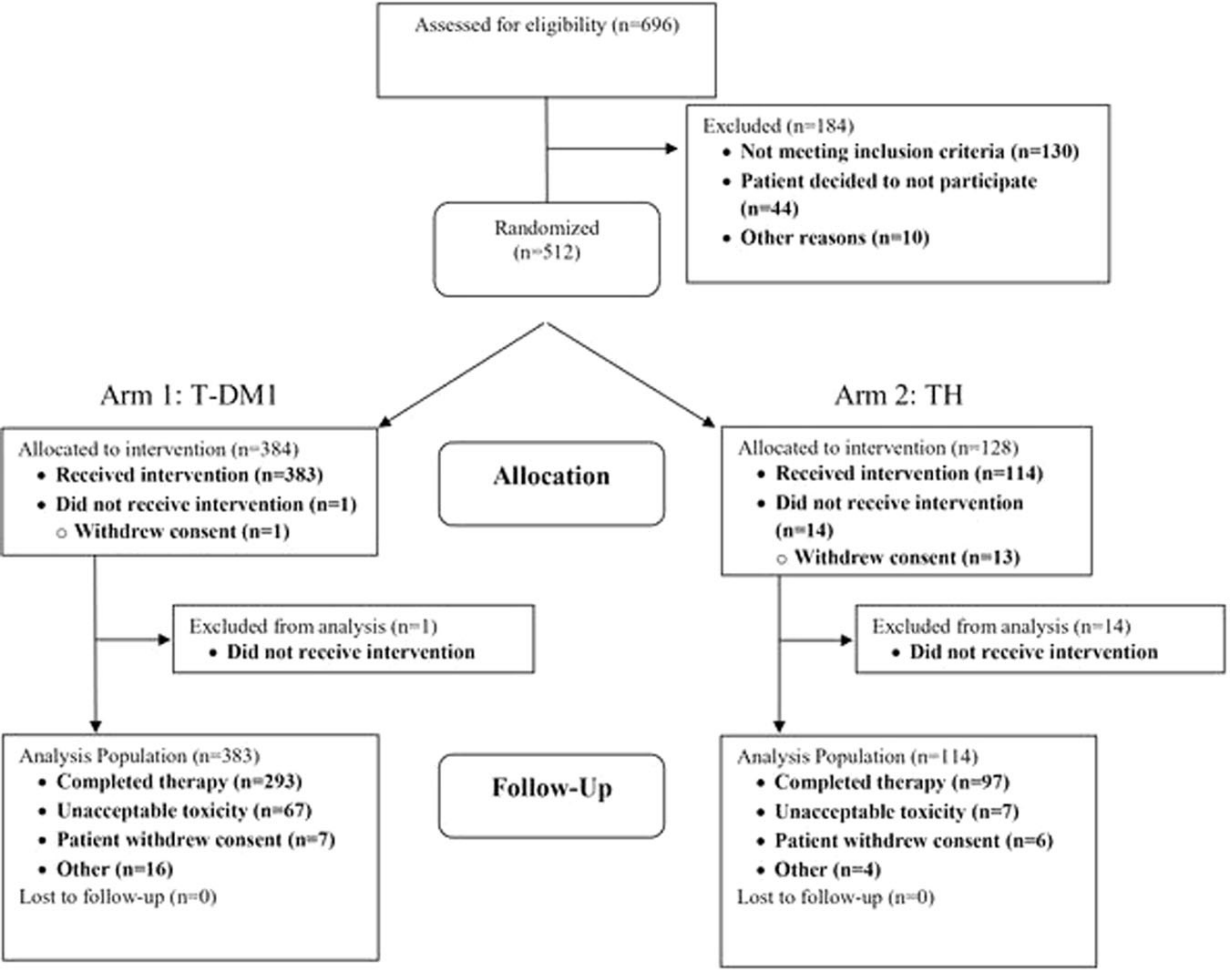
CONSORT Flow Diagram of the study. Among 696 patients assessed for eligibility in the ATEMPT trial, 512 were randomized to receive treatment with either adjuvant T-DM1 (*n* = 384, of which 383 received the intervention) or TH (*n* = 128, of which 114 received the intervention).

From May 17, 2013 to December 13, 2016, 512 patients with stage I HER2-positive breast cancer were enrolled in the ATEMPT trial and 497 (383 T-DM1, 114 TH) started protocol therapy and were included in this analysis (Fig. 1). Over 85% (424 of 497) of patients had baseline left ventricular ejection fraction (LVEF) >55%. Race, ethnicity, and sex were defined by each patient, to demonstrate the enrolled patient population. Median follow-up was 3.9 years, corresponding to 1884 patient-years of follow-up. Baseline patient characteristics did not differ between study arms (Table 1).

**Table 1:** Baseline patient characteristics.

Characteristic	TH arm (*n* = 114)	T-DM1 arm (*n* = 383)
Median age (IQR)	56 [47, 62]	56 [49, 63]
Age group (years)		
<50	41 (36%)	110 (29%)
50−59	35 (31%)	127 (33%)
60−69	30 (26%)	107 (28%)
≥70	8 (7%)	39 (10%)
Sex		
Male	1 (1%)	5 (1%)
Female	113 (99%)	378 (99%)
Race		
White	93 (82%)	327 (85%)
African American	7 (6%)	21 (5%)
Asian	4 (4%)	22 (6%)
Other	10 (9%)	13 (3%)
Ethnicity		
Hispanic or Latino	1 (1%)	11 3%)
Non-Hispanic	97 (85%)	352 (92%)
Ethnicity not known	16 (14%)	20 (5%)
Baseline LVEF		
50–55 (%)	14 (12%)	59 (15%)
>55 (%)	100 (88%)	324 (85%)
BMI		
≤25	42 (37%)	161 (42%)
>25–30	33 (29%)	125 (33%)
>30	39 (34%)	97 (25%)

*BMI* body mass index, *IQR* intraquartile range, *LVEF* left ventricular ejection fraction, *T-DM1* trastuzumab emtansine, *TH* paclitaxel with trastuzumab.

### Changes in left ventricular ejection fraction

Overall, in both arms, the percentage of patients with a decline in LVEF was low (Table 2). In the T-DM1 arm, the fraction of patients with a decline in LVEF of 10–15% from baseline at 3 months, 6 months, 9 months, and 1 year, was 5%, 3%, 3%, and 3%, respectively, and the fraction with an LVEF decline of ≥16% was <1%, <1%, 1%, and <1% at the same timepoints. Similarly, in the TH arm, 5%, 11%, 7%, and 3% of patients had a decline in LVEF of 10–15% from baseline at 3 months, 6 months, 9 months, and 1 year, respectively, and 0, 1%, 0, and 1% had a decline ≥16% from baseline. In both arms, the median LVEF values were preserved throughout treatment (Table 2). When restricting to patients that completed one entire year of treatment, data regarding LVEF changes was consistent with what was observed among the overall study population (Supplementary Table 1).

**Table 2:** Summary of LVEF at protocol-specified time points* and changes from baseline values.

TH arm (*N* = 114)	Baseline	3 months	6 months	9 months	1 year/EOT
LVEF reduction from baseline					
<10%	-	94 (82%)	85 (75%)	86 (75%)	89 (78%)
10–15%	-	6 (5%)	12 (11%)	8 (7%)	3 (3%)
10–15% and below LLN	-	1 (1%)	2 (2%)	1 (1%)	1 (1%)
≥16%	-	0	1 (1%)	0	1 (1%)
Not performed	-	8 (7%)	6 (5%)	7 (6%)	14 (12%)
Not applicable	-	6 (5%)	10 (9%)	13 (11%)	7 (6%)
LVEF level (%)					
Median (IQR range)	62.5 [60–65]	62 [60–65]	60 [59–65]	60 [59–65]	62 [60–65]
T-DM1 arm (*N* = 383)	Baseline	3 months	6 months	9 months	1 year/EOT
LVEF reduction from baseline					
<10%	-	330 (86%)	320 (84%)	284 (74%)	256 (67%)
10–15%	-	19 (5%)	13 (3%)	10 (3%)	12 (3%)
10–15% and below LLN	-	1 (0%)	0	0	1 (0%)
≥16%	-	1 (0%)	1 (0%)	2 (1%)	1 (0%)
Not performed	-	20 (5%)	10 (3%)	21 (5%)	74 (19%)
Not applicable	-	13 (3%)	39 (10%)	66 (17%)	40 (10%)
LVEF level (%)					
Median (IQR range)	63 [60–65]	62 [60–65]	63 [60–66]	63 [60–66]	63 [60–67]

*EOT* end of treatment, *IQR* intraquartile range, *LLN* lower limit of normal, *LVEF* left ventricular ejection fraction, *T-DM1* trastuzumab emtansine, *TH* paclitaxel with trastuzumab.

*Each timepoint uses a window of 6 weeks. If more than one cardiac assessment falls into each window, the worst assessment is used.

### Symptomatic left ventricular systolic dysfunction

Of 497 patients who started protocol therapy, 3 of 383 (0.8%; 95% CI: 0.3–2.3) patients in the T-DM1 arm and 2 of 114 (1.8%; 95% CI: 0.5–6.2) patients in the TH arm developed grade 3 left ventricular systolic dysfunction (LVSD). Among these five patients, only one patient had comorbidities (diabetes mellitus and dyslipidemia) and was receiving concomitant medications for cardiovascular indications. None of these patients had hypertension at baseline. With the exception of one patient who withdrew consent (and for whom we do not have information), all others experienced full resolution of cardiac symptoms and documented LVEF normalization. There was no grade 4 LVSD. Table 3 summarizes the baseline clinical characteristics of these patients, the time of onset of symptomatic cardiac dysfunction and its evolution, and the clinical actions taken following the diagnosis.

**Table 3:** Summary of baseline clinical characteristics and clinical evolution of patients with symptomatic cardiac dysfunction during the ATEMPT study.

#ID	Clinical baseline characteristics				LVEF assessment overtime					Clinical actions after diagnosis of symptomatic cardiac dysfunction and outcome			
	Age	BMI	HTN	DM	BL	3 m	6 m	9 m	End of Tx	Dose hold	Was Tx resumed?	Medication started	Did LEVF normalize^c^?
T-DM1 arm													
12	51	26.4	No	No	60	65	55	30^b^	30	Yes	No	ACEi and beta-blocker	Yes
274	61	21.2	No	No	55	40^b^	NA	NA	40	Yes	No	ACEi	NR
427	65	28.6	No	No	76	58^b^	63	57	57	Yes	No	no	Yes
TH arm													
116	58	21.5	No	No	70	65	58^b^	79	65	Yes	Yes	no	Yes
299^a^	48	39.8	No	Yes	60	60	45^b^	NA	45	Yes	No	Beta-blocker	Yes

*ACEi* angiotensin-converting enzyme inhibitor, *BL* baseline, *BMI* body mass index, *DM* diabetes mellitus, *HTN* hypertension, *LVEF* left ventricular ejection fraction, *M* months, *NR* not reported, *T-DM1* trastuzumab emtansine, *TH* paclitaxel with trastuzumab, *Tx* treatment.

^a^At the time of study start, patient #299 was on metformin and rosuvastatin due to diabetes mellitus and hypercholesterolemia.

^b^Time point when therapy was interrupted.

^c^Among all cases in which LVEF normalized, the symptoms also resolved.

### Incidence of asymptomatic decrease in left ventricular ejection fraction

Three (0.8%) patients in the T-DM1 arm and six (5.3%) patients in the TH arm experienced a significant asymptomatic LVEF decline that per-protocol required trastuzumab hold. Three patients (two in the TH arm and one in the T-DM1 arm) completed treatment before cardiac decline was detected in their last scheduled cardiac function assessment at 12 months. One patient spontaneously recovered LVEF and no clinical action was taken. Another patient was started on losartan and subsequently, her LVEF normalized. Only one patient included in the TH arm discontinued protocol therapy and because the patient withdrew consent, follow-up information is not available. With regards to the other six patients (two in the T-DM1 arm and four included in the TH arm), LVEF spontaneously normalized without the need for any clinical action, and protocol therapy was completed.

### Risk factors for cardiac dysfunction

**Fig. 2:**
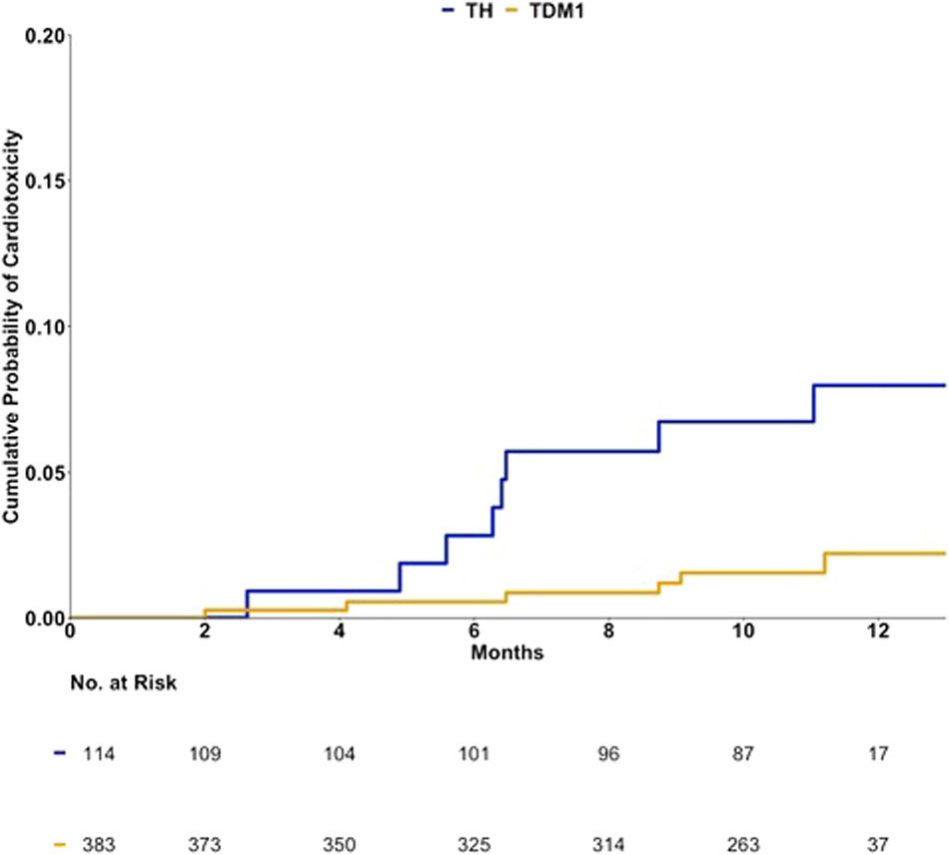
Kaplan–Meier estimate of the cumulative probability of a cardiotoxicity^#^ event during the treatment period. Probability of cardiotoxicity by 6 months: TH: 0.03 (95% CI: 0–0.06); T-DM1: 0.01 (95% CI: 0–0.01). Probability of cardiotoxicity by 12 months: TH: 0.08 (95% CI: 0.02–0.13); T-DM1: 0.02 (95% CI: 0–0.04). Cardiotoxicity here is defined as grade 3–4 left ventricle systolic dysfunction (LVSD) or significant asymptomatic left ventricular ejection fraction (LVEF) decline (decrease in the ejection fraction of 10–15 percentage points from baseline with an ejection fraction at least 1 percentage point below the lower limit of normal, or a decrease of 16 or more percentage points from baseline). T-DM1 trastuzumab emtansine, TH paclitaxel with trastuzumab. *15 patients censored at time 0 due to having only baseline cardiac assessments (ten in T-DM1 arm and five in the TH arm.

We performed an exploratory analysis to assess the relationship between age, baseline LVEF, and body mass index with cardiac outcomes. The percentage of patients with body mass index ≥30 (consistent with obese status) was 34% and 25% the TH and the T-DM1 arms, respectively. No significant association between these baseline characteristics and the incidence of significant asymptomatic LVEF decline or symptomatic LVSD was identified (Table 4). The cumulative probability of having a cardiac dysfunction that ultimately led to T-DM1 interruption or discontinuation at 6 and 12 months was 0.01 (95% CI: 0–0.01) and 0.02 (95% CI: 0–0.04), respectively (Fig. 2). For the TH arm, the cumulative probability of trastuzumab interruption or discontinuation due to a cardiac dysfunction was 0.03 (95% CI: 0–0.06) at 6 months and 0.08 (95% CI: 0.02–0.13) at 12 months.

**Table 4:** Cross-tabulation of baseline characteristics and LVSD/asymptomatic LVEF decline on and off treatment.

LVEF reduction from baseline	Total number of patients	No cardiac toxicity	Symptomatic congestive heart failure or asymptomatic LVEF decline	RR (95%)	*P*
TH arm	114	106	8		
Age at study entry (years)					
<50	41 (36%)	37 (35%)	4 (50%)	Reference	0.42
≥50	73 (64%)	69 (65%)	4 (50%)	0.56 (0.15-2.13)	
Baseline LVEF (%)					
≤55	14 (12%)	13 (12%)	1 (12%)	1.02 (0.14–7.69)	0.92
>55	100 (88%)	93 (88%)	7 (88%)	Reference	
BMI					
≤25	42 (37%)	39 (37%)	3 (38%)	Reference	0.95
>25	72 (63%)	67 (63%)	5 (62%)	0.97 (0.24–3.86)	
T-DM1 arm	383	377	6		
Age at study entry (years)					
<50	110 (29%)	109 (29%)	1 (17%)	Reference	0.58
≥50	273 (71%)	268 (71%)	5 (83%)	2.01 (0.24–17.05)	
Baseline LVEF (%)					
≤55	59 (15%)	57 (15%)	2 (33%)	2.74 (0.51–14.65)	0.28
>55	324 (85%)	320 (85%)	4 (67%)	Reference	
BMI					
≤25	161 (42%)	158 (42%)	3 (50%)	Reference	0.70
>25	222 (58%)	219 (58%)	3 (50%)	0.73 (0.15–3.55)	

*BMI* body mass index, *LVEF* left ventricular ejection fraction, *LVSD* left ventricular systolic dysfunction, *RR* relative risk, *T-DM1* trastuzumab emtansine, *TH* paclitaxel with trastuzumab.

## Discussion

The excellent outcomes observed in patients with early-stage HER2-positive breast cancer treated with (neo)adjuvant HER2-directed therapy have led to successful optimization of regimens with fewer agents in patients with stage I HER2-positive breast cancer^10,11^. More recently, because the outcomes seen in patients treated with adjuvant T-DM1 in the ATEMPT trial and the manageable toxicity profile associated with this agent, it has emerged as a potential alternative therapy to regimens such as TH for select patients with stage I HER2-positive breast cancer^14^. In view of this emerging utilization, as well as its established role as adjuvant treatment for patients with stage II-III breast cancer with residual disease after neoadjuvant therapy, it is of compelling interest to characterize the cardiotoxic profile of T-DM1.

The ATEMPT trial represents a unique opportunity to study cardiac toxicity of T-DM1 as it is the only study that utilized 1 year of T-DM1 without any additional systemic therapies (other than endocrine agents) in the (neo)adjuvant setting. This trial found that T-DM1 is associated with a low rate of grade 3 LVSD with only three patients (0.8%) experiencing a grade 3 event, and two of these patients fully recovered after receiving cardiac medications (the third patient withdrew consent, and her cardiac outcomes are unknown). In the TH arm, there were two patients with grade 3 LVSD (1.8%) and all had full recovery of LVEF. Moreover, all patients with a significant asymptomatic decline in LVEF who would have required treatment interruption per-protocol (0.8% in the T-DM1 and 5.3% in the TH arm) presented with subsequent full recovery of LVEF and completed all planned therapy. Notably, the percentage of patients experiencing a decline in LVEF of 10–15% from baseline never exceeded 5% in the T-DM1 arm, whereas this percentage slightly increased in the TH arm between the third and the 6th month (5 and 11%, respectively), and subsequently dropped to 3% of the patients at 1 year. This trend confirms the reversible nature of trastuzumab-related cardiac toxicity, with permanent events being very rare both with trastuzumab and with T-DM1.

Risk factors associated with trastuzumab-related cardiac toxicity include age, previous anthracycline exposure, coronary artery disease, hypertension, diabetes, smoking, low-normal baseline LVEF (50–55%), and obesity^16,17^. The population in the ATEMPT trial is similar to the one included in the APT study; patients had a median age of 56 years (10% of patients were 70 years or older), and 15% had a low-normal baseline LVEF of ≤55%. We do not have information about all patients’ history of hypertension, diabetes mellitus, coronary artery disease, or smoking. Notably, none of the three patients in the T-DM1 arm who developed grade 3 LVSD had cardiovascular risk factors.

The incidence of significant cardiac adverse events in the T-DM1 arm of the ATEMPT study is similar to what was observed in other studies with different adjuvant regimens without anthracyclines, including data from the APT study^18^ (trastuzumab plus paclitaxel), the BCIRG 006 study^19^ (trastuzumab plus docetaxel and carboplatin), and the phase II study by Jones et al.^20^ with docetaxel and cyclophosphamide with trastuzumab. In these studies, the incidence of grade 3–4 LVSD were ≤0.5%. While the results presented in the current analysis are based on a median follow-up of 3.9 years, it is unlikely that these rates will increase since late cardiac toxicity from HER2-targeting agents is rare^21,22^. In addition, these rates are favorable compared to grade 3–4 LVSD or symptomatic heart failure (HF) rates (2.3%) reported in a pooled analysis of pivotal adjuvant clinical trials using regimens containing anthracyclines^16^. While the incidence of grade 3 LVSD (1.8%) in the TH arm is higher than in the APT study, this could be due to chance given the small number of patients in this arm.

Our study has several limitations. First, data on baseline cardiac risk factors were not uniformly collected in this trial. For instance, we did not collect specific information on the history of hypertension, diabetes mellitus, and coronary artery disease at baseline. In this study, we retrospectively collected this information for patients who developed grade 3–4 LVSD and asymptomatic LVEF decline that led to treatment interruption or discontinuation. Second, LVEF quantifications were not performed according to a prespecified protocol, and testing was conducted per local institutional standards without a central read performed. Third, patients with comorbidities associated with high risk for developing trastuzumab-associated cardiotoxicity, including a history of HF, were excluded from this study, thus these results cannot be generalized to a population of patients with relevant cardiac risk factors. Additionally, the population of patients enrolled in ATEMPT was enriched in white patients, patients younger than 60 years old and with a baseline EF >55%, thus representing a selected population compared to that treated in everyday practice. Lastly, the medical management of decreases in LVEF during therapy was per physician discretion; however, all patients for whom we have information had a full cardiac recovery.

In conclusion, we found in this population of patients with small HER2-positive breast cancers that during treatment with adjuvant T-DM1 the incidence of grade 3–4 LVSD (symptomatic HF) and significant asymptomatic decreases in LVEF were low: both rates were 0.8%. Such low incidence raises the question of whether close serial LVEF monitoring should be performed in all patients or be reserved for patients considered at a higher risk of developing cardiotoxicity and those with symptomatic HF or other cardiac symptoms. Finally, we look forward to seeing the results of ongoing investigations of reduced frequency of LVEF monitoring during anthracycline-free regimens.

## Methods

### Study design and patient population

This was a randomized phase II study across 24 centers in the United States investigating the regimen of weekly paclitaxel with trastuzumab or T-DM1 in patients with stage I HER2-positive breast cancer (NCT01853748). The study was approved by the institutional review board at each site. Written informed consent was obtained from each patient. Patients were required to be ≥18 years of age and have an Eastern Cooperative Oncology Group performance status ≤1, LVEF ≥50%, within 90 days of their most recent breast surgery, and no history of prior breast cancer. Patients were stratified by age (<55 vs. ≥55 years), planned use of radiation therapy (yes/no), and planned use of endocrine therapy (yes/no), and randomized in a 3:1 ratio to receive T-DM1 or TH, respectively. Adjuvant radiation therapy and hormonal therapy (when appropriate) could be initiated after 12 weeks of T-DM1 (Arm 1) or after the conclusion of paclitaxel therapy (Arm 2). Patients in the T-DM1 arm received T-DM1 3.6 mg/kg intravenously on day 1 of each 21-day cycle for a total of 17 cycles or 1 year. Patients on the TH arm received paclitaxel 80 mg/m^2^ intravenously weekly with concurrent trastuzumab, with a loading dose of 4 mg/kg followed by 2 mg/kg administered intravenously, once per week, for 12 weeks. After the completion of 12 weeks of concurrent trastuzumab plus paclitaxel, patients received 6 mg/kg trastuzumab intravenously every 21 days for 13 cycles.

### Study procedures

For this exploratory study, the analysis population was defined as all patients who received any amount of protocol therapy. Assessment of LVEF with echocardiography or multigated acquisition scanning was required at baseline, 3 months, 6 months, 9 months, and 12 months. Interruption of dosing with trastuzumab or T-DM1 was required if a significant asymptomatic decrease of LVEF occurred, here defined as a decrease in the ejection fraction of 10–15 percentage points from baseline with an ejection fraction at least 1 percentage point below the lower limit of normal or a decrease of 16 or more percentage points from baseline. If the ejection fraction did not increase substantially and two consecutive holds of therapy were required, the patient was withdrawn from study treatment. Furthermore, diagnosis of grade 3–4 LVSD during protocol therapy required cessation of trastuzumab therapy. For these patients, we reported the registration date, protocol therapy starting date, off-treatment date, number of cycles administered, and LVEF percentages. Patients who went off treatment early due to grade 3–4 LVSD were also required to have follow-up LVEF assessments 3, 6, and 12 months after the event. All patients with interval development of either symptomatic or asymptomatic LVEF decline described above requiring interruption of trastuzumab or T-DM1 underwent repeat LVEF assessment using the same modality after an interval of 4 weeks. If the LVEF did not recover to a “continue” category as defined by study guidelines and if two consecutive “holds” were required, then the patient would be withdrawn from study treatment. The incidence of grade 3–4 LVSD are secondary endpoints and are the objectives of this current analysis. For these patients, we retrospectively abstracted their medical records searching for baseline information about hypertension, diabetes mellitus, dyslipidemia, coronaropathy, and use of cardiac and antidiabetic medications.

### Statistical considerations

The co-primary objectives of the ATEMPT study were to evaluate invasive disease-free survival in patients receiving T-DM1 and to compare the incidence of clinically relevant toxicities in patients treated with T-DM1 versus TH. These results were previously published.

We analyzed the incidence of grade 3–4 LVSD and significant asymptomatic LVEF decline (previously defined) as binary outcomes. Rates of LVSD and asymptomatic LVEF decline and 95% CIs were calculated using the Wilson method. Time to cardiac dysfunction was assessed using the methods of Kaplan–Meier. The association between risk factors and cardiac dysfunction was assessed using risk ratios, associated 95% Wald confidence intervals, and the exact mid-*p* value. All analyses were conducted using R v3.6.1.

### Reporting Summary

Further information on research design is available in the Nature Research Reporting Summary linked to this article.

## Supplementary information

Supplementary Table 1

NR Reporting Summary

## Data Availability

Individual participant data that underlie the results reported in this article are not able to be shared at this time as patients remain in study follow-up.
