# Supplementary material for: Cardiac outcomes of subjects on adjuvant trastuzumab emtansine vs paclitaxel in combination with trastuzumab for stage I HER2-positive breast cancer (ATEMPT) study (TBCRC033): a randomized controlled trial
Source: NPJ Breast Cancer. 2022 Feb 16;8:18. doi: 10.1038/s41523-022-00385-2 (PMC8850608; doi:10.1038/s41523-022-00385-2)
Supplement: Supplementary file 1 — Supplementary Table 1 [file 41523_2022_385_MOESM1_ESM.pdf]

Supplementary Table 1. Summary of LVEF at protocol specified time points* and changes from baseline values among patients who completed treatment (n=390)

TH arm	Baseline	3 months	6 months	9 months	1 year/EOT
LVEF reduction from baseline					
< 10%	-	86 (89%)	81 (84%)	84 (87%)	84 (87%)
10-15%	-	4 (4%)	10 (10%)	7 (7%)	3 (3%)
10-15% and below LLN	-	0	0	0	0
≥ 16%	-	0	1 (1%)	0	0
Not performed	-	7 (7%)	5 (5%)	6 (6%)	7 (7%)
Not applicable	-	0	0	0	0
LVEF level (%)					
Median (IQR range)	60 [62-65]	60 [62-65]	60 [60-65]	59 [60-65]	60 [62-65]
**T-DM1 arm**	**Baseline**	**3 months**	**6 months**	**9 months**	**1 year/EOT**
LVEF reduction from baseline					
< 10%	-	265 (90%)	272 (93%)	264 (90%)	235 (80%)
10-15%	-	15 (5%)	13 (3%)	10 (3%)	10 (3%)
10-15% and below LLN	-	0	0	0	0
≥ 16%	-	0	1 (0%)	0	1 (0%)
Not performed	-	13 (4%)	11 (4%)	19 (6%)	37 (13%)
Not applicable	-	0	0	0	0
LVEF level (%)					
Median (IQR range)	60 [62-65]	60 [62-65]	60 [62-65]	60 [63-66]	60 [63-67]

EOT, end of treatment; IQR, intraquartile range; LLN, lower limit of normal; LVEF, left ventricular ejection fraction; T-DM1, trastuzumab emtansine; TH, paclitaxel with trastuzumab.
*Each timepoint uses a window of 6 weeks. If more than one cardiac assessment falls into each window, the worst assessment is used.
